# Visual Field Progression in Glaucoma: Comparison Between PoPLR and ANSWERS

**DOI:** 10.1167/tvst.10.14.13

**Published:** 2021-12-15

**Authors:** Iván Marín-Franch, Paul H. Artes, Andrew Turpin, Lyne Racette

**Affiliations:** 1 Computational Optometry, Atarfe, Granada, Spain; 2 Department of Ophthalmology and Visual Sciences, University of Alabama, Alabama, USA; 3 Southwest Eye Institute, Tavistock, UK; 4 Eye and Vision Research Group, Faculty of Health and Human Sciences, University of Plymouth, Plymouth, England, UK; 5 School of Computing and Information Systems, University of Melbourne, Australia

**Keywords:** visual field progression, visual field modeling, sensitivity and specificity, positive rates

## Abstract

**Purpose:**

It has been suggested that the detection of visual field progression can be improved by modeling statistical properties of the data such as the increasing retest variability and the spatial correlation among visual field locations. We compared a method that models those properties, Analysis with Non-Stationary Weibull Error Regression and Spatial Enhancement (ANSWERS), against a simpler one that does not, Permutation of Pointwise Linear Regression (PoPLR).

**Methods:**

Visual field series from three independent longitudinal studies in patients with glaucoma were used to compare the positive rate of PoPLR and ANSWERS. To estimate the false-positive rate, the same visual field series were randomly re-ordered in time. The first dataset consisted of series of 7 visual fields from 101 eyes, the second consisted of series of 9 visual fields from 150 eyes, and the third consisted of series of more than 9 visual fields (17.5 on average) from 139 eyes.

**Results:**

For a statistical significance of 0.05, the false-positive rates for ANSWERS were about 3 times greater than expected at 15%, 17%, and 16%, respectively, whereas for PoPLR they were 7%, 3%, and 6%. After equating the specificities at 0.05 for both models, positive rates for ANSWERS were 16%, 25%, and 38%, whereas for PoPLR they were 12%, 33%, and 49%, or about 5% greater on average (95% confidence interval = −1% to 11%).

**Conclusions:**

Despite being simpler and less computationally demanding, PoPLR was at least as sensitive to deterioration as ANSWERS once the specificities were equated.

**Translational Relevance:**

Close control of false-positive rates is key when visual fields of patients are analyzed for change in both clinical practice and clinical trials.

## Introduction

In patients with glaucoma and other optic neuropathies, changes in the visual field over time are an important marker for deterioration or improvement. Because the goal of any treatment is to preserve visual function, visual field progression is also a key outcome measure in clinical trials of new therapies. As a consequence, techniques for detecting visual field progression have been a focus of research activity for several decades.^1^

It is now well established that global indices, such as mean deviation^2^^,^^3^ are useful for estimating the overall speed of deterioration over time,^4^ but point-by-point analyses — for example, pointwise linear regression,^5^ or glaucoma change probability^6^ — are more sensitive to localized deterioration in the visual field. However, a principal problem with point-by-point analyses is how to estimate the statistical significance of deterioration over the entire visual field. Because the statistical significance may have a direct bearing on the likelihood of making false-positive decisions in clinical care, this is not just a theoretical problem but has real practical importance.

Two recently proposed analyses for localized visual field change are Permutation of Pointwise Linear Regression^7^ (PoPLR) and Analysis with Non-Stationary Weibull Error Regression and Spatial Enhancement^8^^,^^9^ (ANSWERS). Both techniques share many similarities, but ANSWERS attempts to model the distinctive distribution of errors that arise in the estimation of visual field thresholds, whereas PoPLR uses the approximations of least-squares regression.^10^ Moreover, ANSWERS uses population-based cutoff values to derive the statistical significance, whereas PoPLR's *P* value is individualized to each patients' visual field series. The initial papers on ANSWERS have described considerable performance gains over PoPLR. However, it is not clear which of ANSWERS' features contributed most to these gains.

In this study, we investigate the performance of PoPLR and ANSWERS in three independent datasets drawn from longitudinal studies of patients with glaucoma. We demonstrate that the sensitivity differences between PoPLR and ANSWERS are likely driven by differences in specificity. Finally, we discuss some important aspects of progression tests and how these aspects need to be considered in studies comparing between progression tests.

## Methods

### Datasets

Data for analysis were obtained from three longitudinal studies. When both eyes from the same patient were included, one eye was selected at random. Visual fields in each dataset were obtained with the 24-2 SITA-Standard program of the Humphrey Field Analyzer (Carl Zeiss Meditec Inc., Dublin, CA, USA).

The first dataset was selected from the Portland Progression Project (P3), a prospective longitudinal study of patients with early glaucoma (mean deviation [MD] better than −6 dB), suspected glaucoma, or risk factors for the development of glaucoma.^11^ All participants were experienced visual field takers who had performed several tests prior to entry into the study. Additionally, visual fields were removed as unreliable if the percentage of either false positives or false negatives was greater than 20% or if fixations losses were greater than 33%. The percentage of visual fields that did not meet the reliability criteria was less than 3%. Our dataset consisted of 101 eyes of 101 participants with exactly 7 visual fields. The median follow-up period was 3.1 years, with the shorter follow-up period being 2.0 years and the longest 3.8 years. On average, each patient was tested every 5.5 months.

The second dataset was obtained by pooling data from the prospectively designed Diagnostic Innovations in Glaucoma Study (DIGS) and the African Descent and Glaucoma Evaluation Study (ADAGES).^12^ The studies enrolled participants with healthy eyes, as well as glaucoma suspects, and patients with ocular hypertension and primary open angle glaucoma. All visual fields were reviewed for reliability and artifacts by trained graders at the Visual Field Assessment Center at the University of California San Diego.^13^ Briefly, visual fields with more than 33% fixation losses and false-positive errors were excluded. Visual fields with more than 33% of false negative errors were also excluded, except in patients with advanced disease. When a visual field was unreliable, the reading center requested repeat testing when possible. This dataset consisted of 150 eyes of 150 patients with glaucoma with exactly 9 visual fields. The median follow-up period was 4.6 years, with the shorter follow-up period being 2.9 years and the longest 7.5 years. On average, each patient was tested every 6.4 months.

The third dataset was from the Rotterdam Ophthalmic Data Repository (http://www.rodrep.com).^14^^,^^15^ The dataset consisted of visual fields from 139 eyes of 139 patients with manifest glaucoma. In contrast to the previous datasets, the series had a different number of visual fields, with a minimum of 9 and a median of 18. The median follow-up period was 9.3 years, with the shortest follow-up period being 5.2 years and the longest 10.5 years. On average, each patient was tested every 6.3 months. With more than twice as many visits overall and patients who had more advanced glaucoma at baseline (MD = −7.73 dB) than for the P3 (−0.50 dB) and the DIGS/ADAGES (−0.85 dB) datasets, the properties of the Rotterdam dataset were statistically and clinically different. For this dataset, we did not adopt additional selection criteria to remove visual fields depending on patient reliability as false-positive responses, false-negative responses, and fixation losses are not made available.

The Table shows the summary statistics of each of the three datasets used in this study. More details about the datasets and the inclusion and exclusion criteria can be found elsewhere.^11^^,^^12^^,^^14^^,^^15^

**Table.** Summary Statistics of the Three Datasets

	P3 (101 Eyes)			DIGS/ADAGES (150 Eyes)			Rotterdam (139 Eyes)		
	Median	5th	95th	Median	5th	95th	Median	5th	95th
Number of visits	7	7	7	9	9	9	18	15	20
Age at baseline	68	52	79	63	48	76	61	42	72
MD, baseline	−0.50	−8.00	1.23	−0.85	−13.59	0.89	−7.73	−25.65	−1.90
MD, final	−0.68	−9.03	1.31	−1.28	−13.95	0.85	−8.76	−26.72	−2.27
Total MD change	−0.22	−2.65	1.38	−0.30	−3.28	1.92	−0.49	−10.09	3.39
Follow-up time	3.14	2.70	3.61	4.57	3.83	5.97	9.32	7.96	10.10
Rate of MD change	−0.05	−0.96	0.34	−0.05	−0.69	0.33	−0.07	−1.05	0.21

Notes: Age and follow-up time are given in years. MD at baseline and end line are given in decibels (dB). Rate of change is given in dB/year.

Each column shows the median and 5th and 95th percentiles.

MD, Mean Deviation.

### Permutation of Pointwise Linear Regression 

PoPLR^7^ tests the null hypothesis that there is no deterioration anywhere in the visual field. The first step is to compute pointwise linear regression. Thus, for each of the 52 locations of a series of visual fields, a simple linear regression is performed to obtain the corresponding pointwise rate of change and the corresponding *P* value for the one-tailed *t*-test with the alternative that the rate of change is negative. Then, the sum of the natural logarithm of the *P* values in all 52 locations is calculated and its negative value recorded as the *S*-statistic. The PoPLR *S*-statistic equals the one introduced by Fisher^16^ divided by two.

To derive the significance of overall progression in the visual field series, as a whole, PoPLR computes a global *P* value for a significance test based on permutation analysis.^17^ For each visual field series, the value of the observed *S*-statistic is compared with its permutation distribution obtained from 5000 versions of the visual field series that were randomly re-ordered. The *P* value for overall progression of the visual field given the series is obtained as the proportion of random permutations for which the value of the *S*-statistic is greater than for the original series.

The PoPLR analysis is implemented in the open-source package visualFields^18^ (https://cran.r-project.org/web/packages/visualFields/index.html) developed for the R environment for statistical computing.^19^

### Analysis With Non-Stationary Weibull Error Regression and Spatial Enhancement

ANSWERS^8^^,^^9^ is more complex than PoPLR but its fundamental steps are the same: first obtain 52 *P* values for local progression, then combine them computing the *S*-statistic (which was denoted as *I*^−^ in the manuscript introducing the model^8^). The implementation of the ANSWERS model used in this work are detailed exhaustively in the Appendix A.

The two major innovations of ANSWERS are that it takes into account the distinctive distribution of threshold errors as well as the spatial correlations between visual fields locations governed by the anatomic arrangement of the retinal nerve fiber bundles.^20^^,^^21^ The modeling of threshold errors included in ANSWERS aims to tackle the problem that visual field threshold variability increases with depth of defect.^22^^,^^23^

A fundamental difference between PoPLR and ANSWERS is how the *P* value for overall progression for a visual field series is computed: whereas the PoPLR *P* values are based solely on the individual patient's visual field series, the *P* values of ANSWERS are based on significance criteria derived from a reference dataset^24^ (see Fig. S1 in the supplemental material of Zhu et al. 2014^8^). This means that ANSWERS' *P* values are population-based rather than individualized to the specific visual field series.

### Statistical Analyses

First, for the three datasets, we calculated the positive rates of PoPLR and ANSWERS at significance levels (*α*) ranging from *P* < 0.001 to *P* < 0.15; that is, we obtained the proportion of series for which the *P* value derived by PoPLR and ANSWERS was lower than *α*.

Second, we assessed whether the *P* values derived by PoPLR and ANSWERS were accurate. For this, we computed the positive rates for each dataset as in the first analysis, but after randomly re-arranging the time order of the visual fields in each series once. Because the re-ordering is at random, this process is expected to reduce any systematic change in the original series to chance levels. Therefore, the progression rate measured in a sample of re-ordered series equals the false-positive rate, within chance variation. If the *P* values returned by PoPLR and ANSWERS are accurate, the false-positive rate should equal the nominal significance level *α* within sampling error. That is, for *α* = 0.05, the empirically calculated false-positive rate with the re-ordered series should be approximately 5%; for *α* = 0.15, it should be approximately 15%. Because the computation of *P* values with ANSWERS is computationally demanding, we derived the false-positive rates from only one random permutation of each series.

Finally, because the false-positive rate estimates were based on relatively small sample sizes, we carried out an alternative assessment of the accuracy of the *P* values derived by ANSWERS. We derived individualized *P* values with ANSWERS in an approach similar to PoPLR. More precisely, the *P* values were derived by establishing the null distribution of ANSWERS' *S*-statistic from 1000 random permutations of each original visual field series. By design, this approach ensures close control over the false-positive rate and the accuracy of the *P* values. Positive rates were then obtained for this modified ANSWERS model as in the first analysis. Because ANSWERS is computationally highly demanding this became practicable only through use of the high-performance computing facilities at the University of Melbourne.

## Results

Our implementation of ANSWERS failed to converge in 2 out of the 101 eyes of the P3 dataset and 6 out of the 139 eyes from the Rotterdam dataset. The eight eyes that could not be analyzed with ANSWERS were removed from the study. Thus, the sample sizes for the subsequent analyses were 99 eyes for the P3 dataset, 150 eyes for the DIGS-ADAGES dataset, and 133 eyes for the Rotterdam dataset.

**Figure 1.**
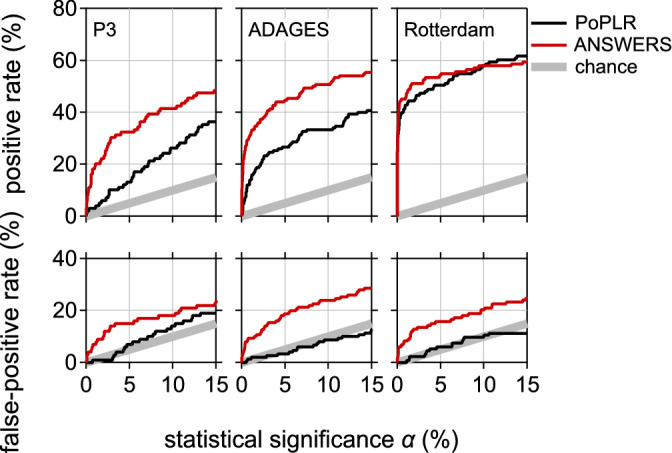
**Positive rates and false-positive rates as a function of the nominal statistical significance set for deterioration analyses with PoPLR and ANSWERS**. Each panel shows the positive rates obtained at different statistical significance values *α* expressed as a percentage. The lower panels show the same as the upper panels but after randomly re-ordering the dates in each series.

The upper panel of Figure 1 shows the positive rates for PoPLR and ANSWERS for the three datasets. The lower panel of Figure 1 shows the false-positive rates calculated after randomly re-ordering the visual fields in each series.

**Figure 2.**
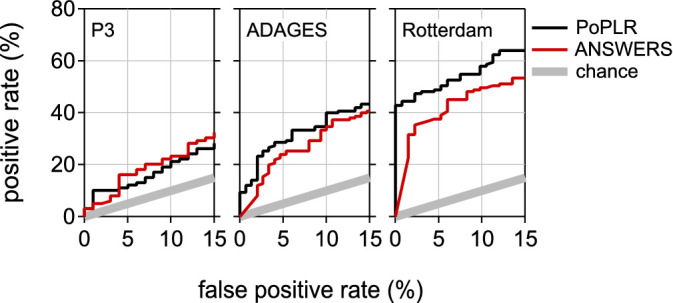
**Positive rates as a function of the false-positive rates for PoPLR and ANSWERS.** Details as for Figure 1. The data in the *y*-axes in these graphs are the same as for the *y*-axes in the upper panel's graphs of Figure 1; the data in the *x*-axes are the same as for the *y*-axes in the lower panel's graphs of Figure 1.

The positive rates for ANSWERS were clearly greater than for PoPLR, as shown in the upper panels of Figure 1. But, as shown in the lower panel, so were the false-positive rates. To correct for this disparity in false-positive rates and thus allow for a fair comparison of the positive rates obtained with the models, Figure 2 shows the positive rate (*y*-axes in the uppers panel of Fig. 1) as a function of the false-positive rates (*y*-axes in the lower panels of Fig. 1).

For all three datasets, positive rates for PoPLR were similar or greater than those for ANSWERS.

**Figure 3.**
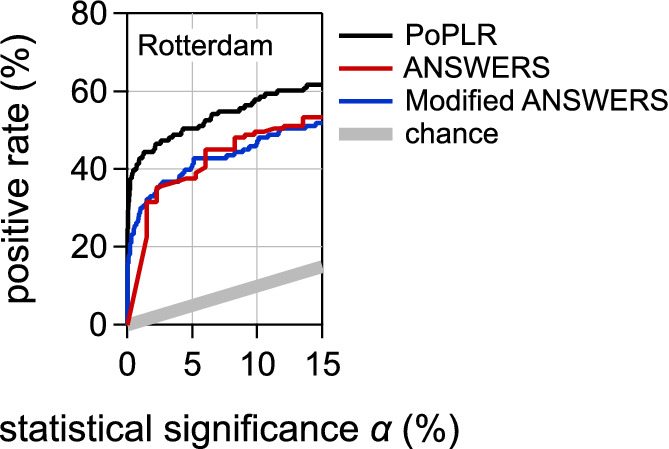
**Positive rates for PoPLR, ANSWERS, and modified ANSWERS as a function of the statistical significance.** The curves for PoPLR and ANSWERS here are the same as for the upper-right panel of Figure 2. Other details as for Figure 1.

To confirm these findings, we recomputed *P* values using 1000 random visual field permutations with ANSWERS. Figure 3 shows the positive rates obtained for random permutation with this modified version of ANSWERS for the Rotterdam dataset. For comparison, Figure 3 also shows the positive rate obtained for PoPLR and for ANSWERS as a function of the false-positive rate (the black and red curves in the right panel of Fig. 2).

The positive rates obtained for random permutation with the modified ANSWERS (blue curve) matched those for ANSWERS expressed as a function of its false-positive rates (red curve). Positive rates were similar to each other and always clearly lower than for PoPLR.

## Discussion

The conception of ANSWERS^8^^,^^9^ is grounded in sound ideas based on the well-documented non-Gaussian and heteroscedastic properties of visual field threshold estimates,^22^^,^^23^^,^^25^ as is the inclusion of spatial correlations among visual field locations.^20^^,^^21^ Nevertheless, comparisons against PoPLR,^7^ which uses simple linear regression and ignores non-Gaussian heteroscedastic errors and spatial correlations, do not support the notion that these features have a large impact on the sensitivity to visual field deterioration.

For ANSWERS, the false-positive rates obtained from the randomly re-ordered series (see Fig. 1, lower panel) were much larger than the nominal significance values. This suggests that the specificity of ANSWERS is lower than intended, and consequently that the positive rates of ANSWERS (see Fig. 1, upper panel) are inflated. In other words, ANSWERS' substantial improvement of sensitivity to progression in comparison to PoPLR comes at the cost of a reduced specificity. For a statistical significance level of 5% (corresponding to a specificity of 95%), the false-positive rate was 15% for the P3 dataset, 19% for DIGS-ADAGES dataset, and 16% for the Rotterdam dataset —3 times greater than expected.

A possible reason for ANSWERS' lower-than-expected specificity, and the resulting overestimation of sensitivity, lies in ANSWERS' way of obtaining the *P* values from the *S*-statistics. To derive its *P* value, ANSWERS compares an individual patient's *S*-statistic to criteria obtained from a reference group of patients with glaucoma. This is problematic for two reasons. First, the salient properties of visual field series (e.g. variability and distribution of visual field damage) differ vastly between patients, and therefore a population statistic is an imperfect yardstick for judging the significance of change in an individual. For a given amount of change, it will underestimate the significance in patients who are more fastidious visual field takers compared to the average patient, and it will overestimate significance in poor observers.^26^ So, even if the significance criteria were derived from a perfectly representative sample such that the calibration of *P* values were accurate on average, these *P* values could still be misleading in individuals who differ from the group average.

Second, it is not easy to select a reference population that is representative of the target population. Clearly, criteria for change derived from patients who are highly experienced and enthusiastic participants in visual field studies (e.g. the reference group^24^ used by the authors of ANSWERS) are unlikely to be sufficiently conservative to ensure the desired level of specificity in clinical groups of patients (e.g. the three datasets used in this study). This may be the most compelling explanation for the lower specificity of ANSWERS in this study compared to the original publications.^8^^,^^9^ Therefore, individualized significance-of-change approaches (as used in PoPLR) are preferable to population-based criteria. By controlling the specificity at the level of the individual patient, we can be confident that the specificity at the population level is closely controlled also.

The Glaucoma Progression Analysis (GPA) of the Humphrey Field Analyzer is a widely used approach for investigating visual field changes in clinical practice. It is based on no-change intervals derived from a group of stable patients. Through its use in the Early Manifest Glaucoma trial,^6^ as well as in the United Kingdom Glaucoma Treatment Study,^27^ the GPA is supported by solid clinical evidence. However, the limitations of population-based change criteria apply equally to the GPA: some individuals are much more likely to show significant change than are others.^24^ Although ways have been suggested to amend the issue,^28^ we believe that permutation analysis of individual visual field series (as in PoPLR) may provide a more comprehensive solution.

We found that not all visual field series could be analyzed by ANSWERS. The *S*-statistic could not be computed in 3224 series (2.3%) out of 139,000 permutations (139 series × 1000 permutation per series) performed to generate Figure 3. The inability to return a valid result in a small proportion of visual field series was due to failures in convergence of the optimization algorithm. The algorithm searches for the optimal value of 104 parameters (intercept and slope for each of 52 locations) and sometimes only a suboptimal result (a local maximum) is achieved and the estimated standard errors are unreliable. Because the standard errors are required to compute *P* values, the model can break down. Zhu et al. did not report on failures to fit the model, and there are minor differences between our implementation and the original one (see Appendix A). This motivated us to share our implementation in the supplementary computer code, so that it can be critically evaluated by the community. In addition, differences with respect to the original implementation described by Zhu and colleagues,^8^^,^^9^ and an example replicated from the authors' manuscript, are described and discussed in detail in the Appendix A.

Zhu and colleagues^8^^,^^9^ developed a similar version of the analysis without spatial enhancement (ANSWER) and found that positive rates were smaller than with spatial enhancement (ANSWERS). We found a similar result (see Supplementary Fig. S5 in Appendix B). However, these differences in performance vanished (see Supplementary Fig. S7 in Appendix B) if separate cutoff values to compute *P* values from *S*-statistics are generated specifically for ANSWER, as shown in Supplementary Figure S6 in Appendix B.

In summary, our findings demonstrate that the initially reported differences in sensitivity between ANSWERS and PoPLR are largely explained by differences in specificity, and that the performance differences between the two approaches are minor once specificity is equalized. Our study also illustrates the need to replicate performance evaluations with other, independent datasets, and we are indebted to our colleagues who made longitudinal visual field data available for such work. Replication of visual field progression analyses becomes easier when the source code is made freely available and open to be used, scrutinized, and improved by the community. Publishing code and sample scripts along with the initial papers should become standard practice in visual field research as it already is in other mature disciplines.

## Supplementary Material

Supplement 1

Supplement 2
