# Supplementary material for: Visual Field Progression in Glaucoma: Comparison Between PoPLR and ANSWERS
Source: Transl Vis Sci Technol. 2021 Dec 15;10(14):13. doi: 10.1167/tvst.10.14.13 (PMC8684309; doi:10.1167/tvst.10.14.13)
Supplement: Supplement 1 [file tvst-10-14-13_s001.pdf]

Appendix A. Implementation of ANSWERS

The model ANSWERS proposed by Zhu et al [1] was re-implemented in R [2]. As with any implementation, there are some differences between our implementation and that of Zhu et al. [1, 3]. We used the same dataset [4] as in the PLoS ONE implementation [1]. The dataset (available in the open source visualFields package [5] for R) consists of 30 eyes of 30 patients with different degrees of glaucoma severity, each tested 12 times with static automated perimetry over a period of 3 months. Figure S1 shows the conditional retest distribution.

From the series of visual fields for all 30 eyes, we generated a retest dataset of 1980 pairs of repeated visual fields using combinations as in [1]. We then obtained the non-stationary Weibull error mixture distributions for sensitivities from 0 dB to 35 dB. Figure S2 shows the retest histograms and Weibull mixture fits (red curves). Table S1 shows the values of the parameters for the Weibull mixture that we obtained for each sensitivity level. Although we followed the procedures set in [1] to the best of our ability, some of the fits obtained were clearly suboptimal and we needed to repeat the fitting procedure until we obtained the reasonable fits shown in Figure S2. We used a ceiling at 40 dB, to ensure the range of retest variability was between 0 dB and 40 dB.

Our Weibull fits differed from those shown in Figure 3 of [1], even though the same dataset was used. Remarkably, for 0 dB, the probability to get a retest sensitivity level of 0 dB is about 0.4 according to Zhu et al's histograms and Weibull fits, whereas it is almost twice as probable, 0.7, according to our analysis. In Figure 3 of [1], each bar in the histograms

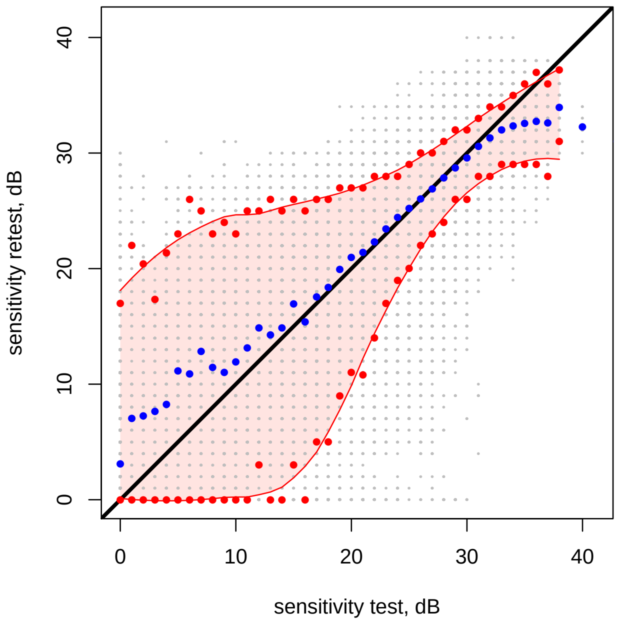

Figure S1: Retest conditional distribution obtained with the test-retest dataset [4]. Each dot represents sensitivity values obtained for the retest at each sensitivity value in the test. The red dots represent the empirical 5th and 95th quantiles and the curves are fits obtained with loess. The black solid line is the 1-to-1 line and the blue dots are the medians.

corresponds to two sensitivity levels; that is, there is a total of 20 bars for 40 dB levels. This binning has a smoothing effect in the data that likely explains the disagreement between

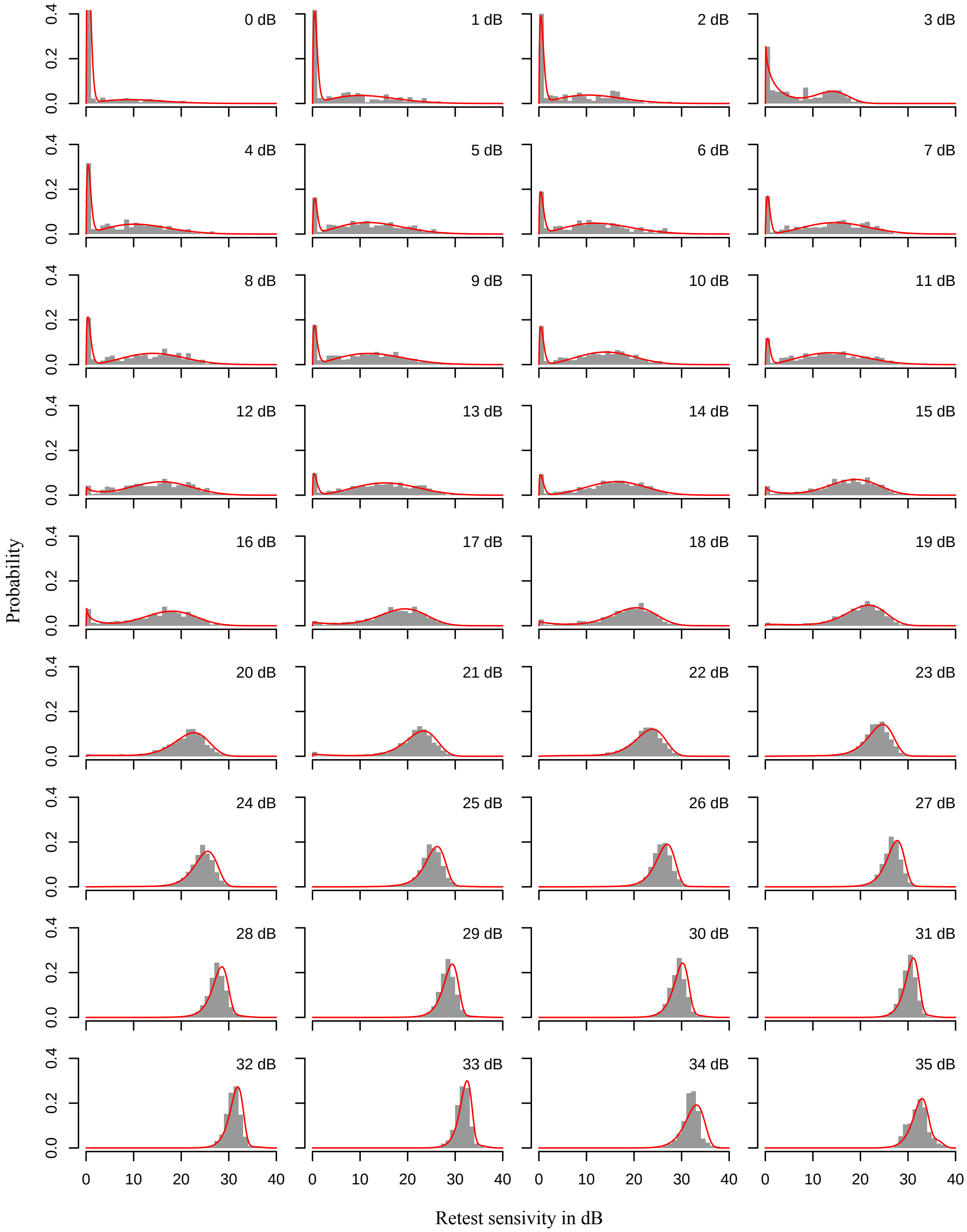

Figure S2: Relative frequencies of retest sensitivities at sensitivity levels from 0 to 35 dB. In each graph, the fitted Weibull mixture with the parameter values as shown in Table S1 is superimposed in red. Notice that for the sensitivity level of 0 dB, the bar and value of the Weibull mixture fit at zero are greater than 0.4. The relative frequency for a retest value of 0 dB was about 0.7.

histograms and fits.

Table S1: Parameters for the non-stationary Weibull error mixture distributions for eq. (4) from [1] and using the same notation. The fits are shown in Figure S2. For clarity, the parameters in this table are presented so that $\pi_1 > \pi_2$, otherwise, the parameters have the same values as in the visualFields package [5].

dB	π_1	α_1	β_1	π_2	α_2	β_2
0	0.74	1.63	0.80	0.26	1.96	12.91
1	0.57	2.05	13.89	0.43	1.61	0.80
2	0.58	2.13	13.91	0.42	1.55	0.80
3	0.56	0.87	2.79	0.44	4.67	14.99
4	0.67	2.13	13.72	0.33	1.59	0.80
5	0.84	2.25	15.04	0.16	1.66	0.80
6	0.80	2.25	15.37	0.20	1.49	0.80
7	0.83	2.70	17.39	0.17	1.69	0.80
8	0.77	2.72	16.62	0.23	1.49	0.80
9	0.81	2.26	15.25	0.19	1.52	0.80
10	0.82	2.82	16.31	0.18	1.54	0.80
11	0.88	2.51	16.84	0.12	1.67	0.80
12	0.88	3.10	18.18	0.12	0.87	4.34
13	0.89	2.76	17.91	0.11	1.42	0.80
14	0.90	3.17	18.40	0.10	1.44	0.80
15	0.89	4.16	20.31	0.11	0.85	4.08
16	0.84	3.91	19.49	0.16	0.81	2.91
17	0.90	4.53	20.62	0.10	1.03	6.50
18	0.90	5.03	21.44	0.10	0.91	5.97
19	0.93	5.86	22.42	0.07	1.11	8.66
20	0.93	7.00	23.21	0.07	1.28	11.31
21	0.93	7.71	23.76	0.07	0.91	7.96
22	0.94	8.38	24.27	0.06	1.71	14.61
23	0.95	10.02	24.94	0.05	2.19	19.41
24	0.96	11.48	25.79	0.04	2.19	19.97
25	0.90	13.77	26.35	0.10	4.81	25.60
26	0.95	14.51	27.10	0.05	3.78	25.73
27	0.95	16.20	27.83	0.05	5.02	27.67
28	0.87	18.94	28.61	0.13	8.28	29.37
29	0.93	19.99	29.46	0.07	7.48	30.52
30	0.89	21.22	30.27	0.11	10.17	31.42
31	0.91	23.63	31.18	0.09	12.59	32.49
32	0.95	24.34	31.94	0.05	12.38	33.91
33	0.91	27.97	32.53	0.09	14.24	33.97
34	1.00	17.33	33.25	0.00	27.17	19.95
35	0.85	21.96	32.93	0.15	22.44	35.88

We used the optic-nerve-head angles of incidence for each visual field location as specified in Figure 4 in [6] and the eq. (8) in [1] to derive the spatial correlations between locations. Figure S3 shows grayscale representing spatial correlations. There seem to be differences between our spatial correlations and those in Figure 2 in [1]. The differences could be that they did use different OCT angles from those in Figure 4 in [6] or just a difference in the graphical display of the spatial-correlation data.

Fits for a series of visual fields consisted on maximizing the logarithm of the posterior probability, see eq. (9) in [1]. The posterior probability can be manipulated as to consider the spatial correlations shown in Figure S3 (ANSWERS) or not (ANSWER). Notice that there is a typographical error in the eq. (9): from eq. (6), it follows that the right hand side of the equality ought to start with either $\sum_{i=1}^{N}\sum_{j=1}^{M}\ln R_{y_{ij}}(\cdot)$ or $\ln\prod_{i=1}^{N}\prod_{j=1}^{M} R_{y_{ij}}(\cdot)$, where $R_{y_{ij}}(\cdot)$ is defined in eq. (4).

For the 24–2 test, ANSWERS attempts to obtain the values of 104 parameters (52 slopes

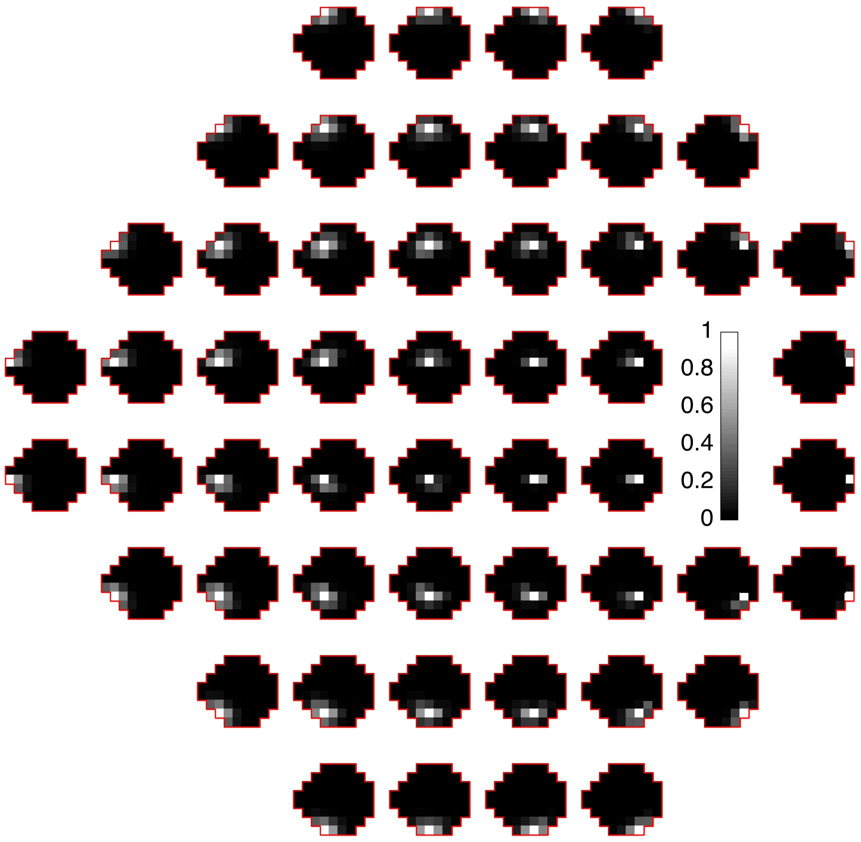

Figure S3: Spatial correlation between each location and all other locations in the visual field.

and 52 intercepts for 52 locations, after excluding those affected by the blind spot) that maximizes the logarithm of the posterior probability. As in any optimization algorithm, the 104 parameters need to be given an initial value so that the logarithm of the posterior probability can be computed and compared against that computed for other values of the parameters. The optimization algorithm then iteratively modifies the values of the 104 parameters until the logarithm of the posterior probability does not increase any more. At each of the 52 locations, we used the median over all sensitivity values in the series for that location as the initial estimates for the intercept and zero as the initial estimates for the slope, as in [1]. Note that as with any optimization algorithm, convergence to the global maxima of the logarithm of the posterior probability is not ensured. Convergence of the algorithm only ensures that a local maxima is reached. If other values are used to initialize the 52 intercepts and 52 slopes, the algorithm may converge to another local maxima, or the global maxima, for which the logarithm of posterior probability is even greater.

Once the 52 intercepts and 52 slopes have been estimated with the optimization algorithm to maximize the logarithm of the posterior probability, the probability of no deterioration is also estimated. Zhu and colleagues used the Laplace approximation (explained in the supplemental material S1 of [1]) to approximate the posterior probability with a Gaussian distribution with a covariance matrix also approximated by the inverse of the negative Hessian

matrix. From the covariance matrix and for each of the 52 locations, the estimated standard deviation for the slope at that location can be extracted to calculate the probability that the "true slope" is greater than 0, which Zhu et al called *probability of no deterioration*. But this probability of no deterioration equals the p-value of a 1-tail significance test that slope equals zero versus slope is lower than zero. In more detail, let s be the theoretical "true slope" for a location, and $\hat{s}$ and $\hat{\sigma}$ the slope and standard deviation estimated with ANSWERS. Then, slope s follows a Gaussian distribution with mean $\hat{s}$ and standard deviation $\hat{\sigma}$. On the other hand, if the null hypothesis is true, then the null distribution is Gaussian with zero mean and standard deviation $\hat{\sigma}$. Let s_0 be a random variable for the slope under the null hypothesis. Then, the p-value is defined as the probability of obtaining a slope value $\hat{s}$ or smaller. Since $s = s_0 + \hat{s}$, then

$$P\{s_0 \leq \hat{s}\} = P\{\hat{s} - s \leq \hat{s}\} = P\{-s \leq 0\} = P\{s \geq 0\}\,.$$

The left-hand side of the sequence of probability equivalences is the p-value and the right-hand side is the probability of no deterioration.The probability of no deterioration and the p-value of the 1-tail significance test for slope are two sides of the same coin.

Despite the differences in the implementation, we obtained remarkably similar fits to those shown in Figure 4 in [1]. Figure S4 shows the fits we obtained using the same data as in Figure 4 in [1]. The slopes with simple linear regression were -2.4 dB per year (p-value $= 0.133$) for the example shown in the left panel and -1.6 dB per year (p-value $= 0.071$) for the example shown on the right panel. The slopes obtained with ANSWERS were 0 dB per year (p-value $= 0.52$) and -1.1 dB per year (p-value $= 0.046$).

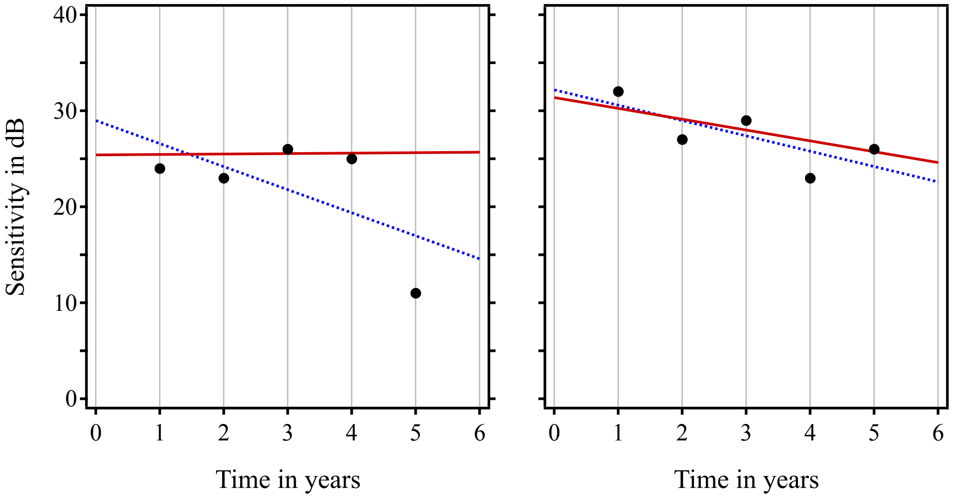

Figure S4: Two examples comparing simple linear regression and ANSWERS. The solid black circles are sensitivity values measured 1 year apart. They were digitized from Figure 4 in [1]. The blue dotted lines show the linear fits obtained with simple linear regression and the red solid lines show the ANSWERS linear fits.

The slopes and intercepts obtained with the re-implementation of ANSWERS used in this work depend heavily on the year of origin, perhaps a consequence of the use of a prior

probability for intercepts, from which the posterior probability to maximize is dependent on. That is, the slope estimated for the same sequence of sensitivity values (e.g., $32, 27, 29, 23, 26$ as for the example in the right panel of Figure S4) would differ if the time in years in the x axis starts at zero, instead of one. In fact, the estimated slope changed from -1.1 dB per year (p-value $= 0.046$) to -1.2 dB per year (p-value $= 0.039$). If it started at 2 years, the slope would be -1.1 dB per year (p-value $= 0.055$), no longer significantly smaller than zero. In this re-implementation of ANSWERS, all time series were shifted to start at year 0, which seemed the most natural decision given the definition of the posterior probability.

Once the 52 p-values (or probabilities of no progression) for slopes have been estimated for a series of visual fields, the results can be summarized into a single statistic to assess if there is evidence of deterioration in the visual field as a whole. Fisher's method [7] is used by ANSWERS, a generalization of which (the truncated product method [8]) is used in PoPLR [9]. For consistency and simplicity, the statistic obtained with Fisher's method is denoted here by S, instead of by I^- as in [1]. The statistic S is calculated from all p-values for all 52 locations p_i; $i = 1, \cdots, 52$ as

$$S = -\sum_{i=1}^{52} \log p_i \,,$$

where log represents the natural logarithm. The S-statistic is used by both PoPLR and ANSWERS to test the hypothesis that the visual field is stable vs the hypothesis that is deteriorating with a 1-tail significance test. The PoPLR method [9] calculates statistical significance for visual field deterioration individualized to the particular patient's data with permutation analysis [10]. But for ANSWERS, Zhu et al [1] obtained threshold values for S by applying permutation analysis in the retest dataset [4], instead of using individual subject's data. They used a different dataset in [3]. Here, the same procedures as in [1] with the retest dataset were used: for each eye, the series of 12 visual fields was randomly reordered 300 times to generate a total of 90 000 series of lengths between 3 and 12 visual fields. And it was assumed that the visual fields were taken at a rate of one per year.

There were small differences between the quantile functions obtained here for ANSWERS and those shown in Figure S1 in the supporting material of [1]. For instance, the value of S for 0.05 obtained here was 56.2, whereas it was 57.5 in [1]. To assess the impact of that difference in S, imagine that the p-values obtained for all 52 locations with ANSWERS are equal to each other and equal to p. Then $p = \exp(-S/52)$, a value of 56.2 corresponds to $p = 0.339$ and a value of 57.5 to $p = 0.331$. Thus, the differences in S quantiles correspond to small differences in p-value. Therefore, despite the differences in Weibull mixture fits and spatial correlations and the optimization algorithms used internally, both the fits (see Figure S4) and the quantile functions obtained here are remarkably similar to those in [1].

Appendix B. Differences between ANSWERS and ANSWER

Zhu et al developed two versions of the model [1, 3], one with spatial enhancement (ANSWERS) and one without spatial enhancement (ANSWER). They found differences, with ANSWERS generally yielding greater positive rates, particularly for shorter series of visual

fields (see Figure 6 of Zhu et al [1] and Figure 2 of Zhu et al [3]). We found similar differences between ANSWER and ANSWERS as shown in Figure S5.

During the implementation of ANSWERS, we noticed that the empirical quantiles obtained with ANSWER were clearly different as those obtained with ANSWERS with the same reference retest dataset [4]. Figure S6 shows empirical quantiles obtain for ANSWERS (left) and ANSWER (right) for series of 3 to 12 visual fields. A S of 56.2 obtained with

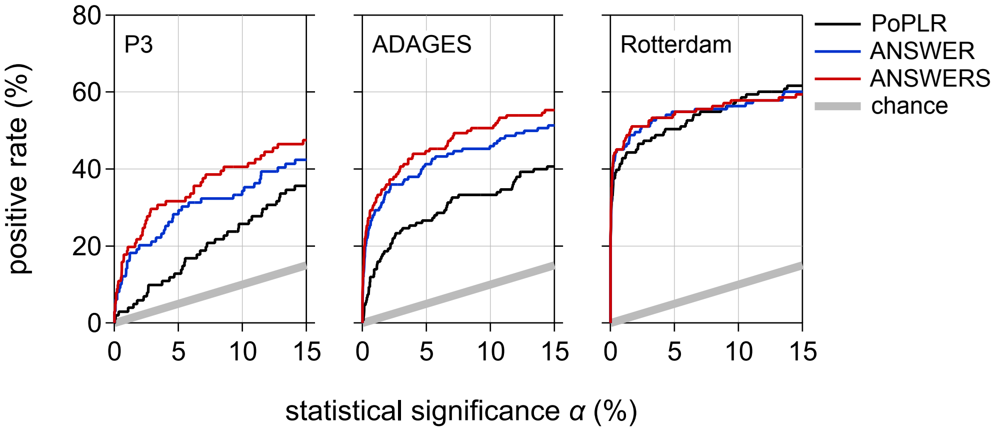

Figure S5: Replication of the upper panel in Figure 1 including results the results of ANSWER.

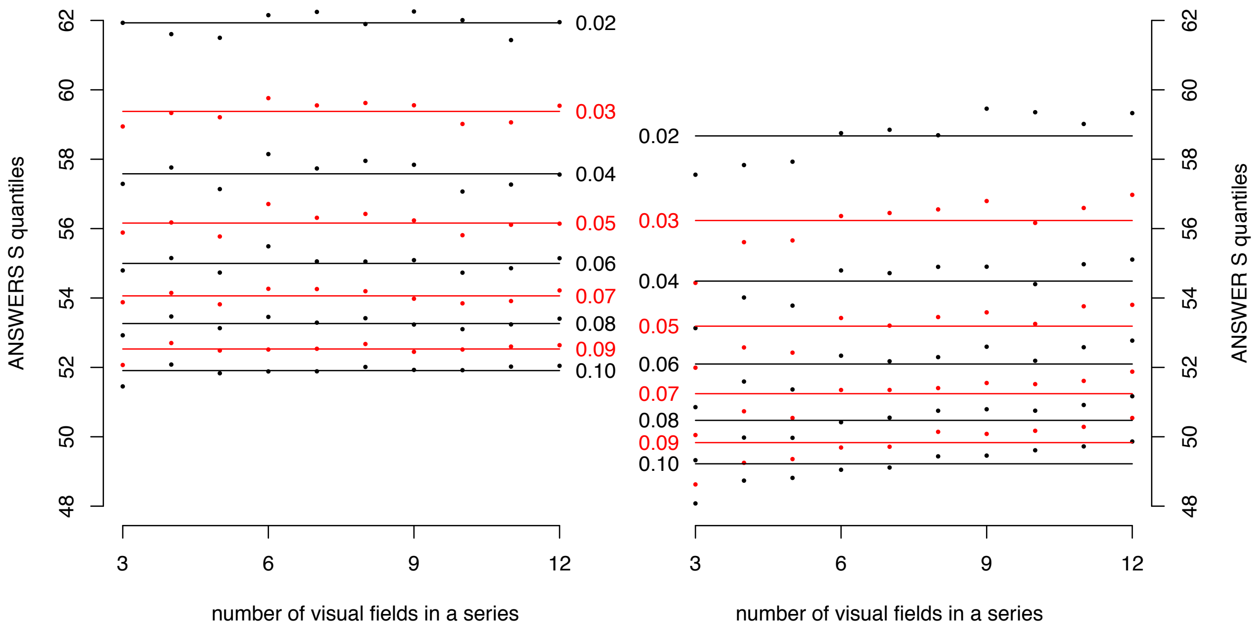

Figure S6: Empirical quantiles (solid circles) for the S-statistic and quantile functions (horizontal lines) for series of 3 to 12 visual fields. The fitted quantile functions are simply the average empirical quantile for the S value over the ten series lengths. For clarity, solid curves and fitted lines in each graph are shown in black or red color intercalated.

ANSWERS and of 53.2 with ANSWER would be assigned the same p-value of 0.05. If other words, if the same look-up table is used for both ANSWERS and ANSWER then, then by design, ANSWERS would systematically give smaller p-values and it would appear to be more sensitive than ANSWER. To illustrate this point, consider the look-up table for ANSWERS (right of Figure S6) to be the reference for ANSWER too. At a specificity of 95%, any eye with $S > 56.2$ would be flagged as progressing with ANSWERS. The p-value corresponding to the cutoff of 56.2 looking at ANSWER look-up table (right) is actually 0.03 for ANSWER, corresponding to a specificity of 97% or 2% greater than for ANSWERS.

The differences found Figure S5 and in Zhu et al [1, 3] between ANSWERS and ANSWER vanished once the p-values were obtained with the look-up table corresponding to each model, as shown in Figure S7.

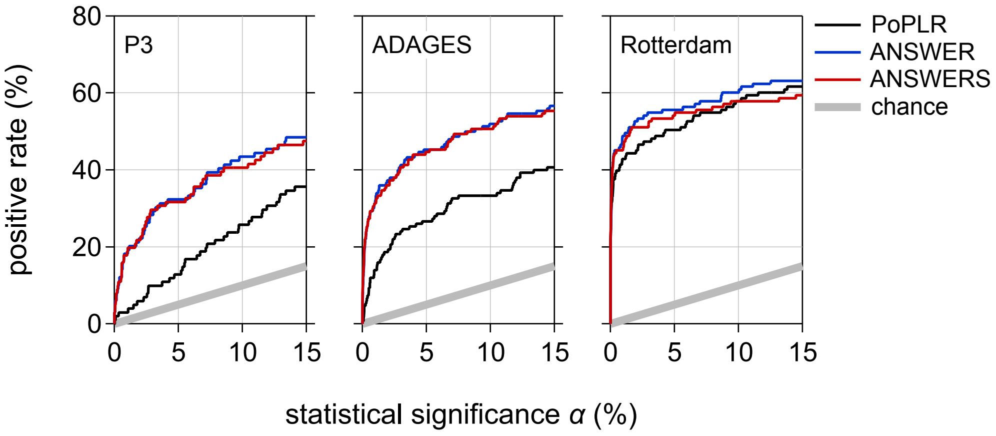

Figure S7: Replication of Figure S5 where the look-up table for mapping S-statistics to p-values is different for ANSWER than for ANSWERS.

References

[1] H. Zhu, R. A. Russell, L. J. Saunders, S. Ceccon, D. F. Garway-Heath, and D. P. Crabb. Detecting changes in retinal function: Analysis with non-stationary Weibull error regression and spatial enhancement (ANSWERS). *PLoS One*, 9(1):e85654, 2014.

[2] R Core Team. *R: A Language and Environment for Statistical Computing*. R Foundation for Statistical Computing, Vienna, Austria, 2021.

[3] H. Zhu, D. P. Crabb, T. Ho, and D. F. Garway-Heath. More accurate modeling of visual field progression in glaucoma: ANSWERS. *Investigative Ophthalmology and Visual Science*, 56:6077–6083, 2015.

[4] P. H. Artes, N. O'Leary, M. T. Nicolela, B. C. Chauhan, and D. P. Crabb. Visual field progression in glaucoma: What is the specificity of the guided progression analysis? *American Academy of Ophthalmology*, 121(10):2023–2027, 2014.

[5] I. Marín-Franch and W. H. Swanson. The visualfields package: A tool for analysis and visualization of visual fields. *Journal of Vision*, 13(4):10,1–12, 2013.

[6] D. F. Garway-Heath, D Poinoosawmy, F. W. Fitzke, and R. A. Hitchings. Mapping the visual field to the optic disc in normal tension glaucoma eyes. *American Academy of Ophthalmology*, 107(10):1809–1815, 2000.

[7] R. A. Fisher. *Statistical Methods for Research Workers.* Oliver and Boyd Ltd., Edinburgh: Tweeddale Court, London, fifth edition, 1934.

[8] D. V. Zaykin, L. A. Zhivotovsky, P. H. Westfall, and B. S. Weir. Truncated product method for combining p-values. *Genetic Epidemiology*, 22:170–185, 2002.

[9] N. O'Leary, B. C. Chauhan, and P. H. Artes. Visual field progression in glaucoma: estimating the overall significance of deterioration with permutation analyses of pointwise linear regression (PoPLR). *Investigative Ophthalmology and Visual Science*, 53(11):6776–84, 2012.

[10] P. I. Good. *Permutation, Parametric, and Bootstrap Tests of Hypotheses.* Springer Series in Statistics. Springer, 3rd edition edition, 2005.
